# Influences of glutamine administration on response selection and sequence learning: a randomized-controlled trial

**DOI:** 10.1038/s41598-017-02957-w

**Published:** 2017-06-02

**Authors:** Bryant J. Jongkees, Maarten A. Immink, Lorenza S. Colzato

**Affiliations:** 1 Institute of Psychological Research and Leiden Institute for Brain and Cognition, Leiden University, Leiden, The Netherlands; 2 School of Health Sciences & Cognitive Neuroscience Laboratory, University of South Australia, Adelaide, Australia; 3 Institute of Cognitive Neuroscience, Faculty of Psychology, Ruhr University Bochum, Bochum, Germany; 4 Institute for Sports and Sport Science, University of Kassel, Kassel, Germany

## Abstract

Precursors of neurotransmitters are increasingly often investigated as potential, easily-accessible methods of neuromodulation. However, the amino-acid glutamine, precursor to the brain’s main excitatory and inhibitory neurotransmitters glutamate and GABA, remains notably little investigated. The current double-blind, randomized, placebo-controlled study provides first evidence 2.0 g glutamine administration in healthy adults affects response selection but not motor sequence learning in a serial reaction time task. Specifically, glutamine increased response selection errors when the current target response required a different hand than the directly preceding target response, which might indicate enhanced cortical excitability via a presumed increase in glutamate levels. These results suggest glutamine can alter cortical excitability but, despite the critical roles of glutamate and GABA in motor learning, at its current dose glutamine does not affect sequence learning.

## Introduction

There is growing research interest in evaluating the neuromodulatory effects of exogenous administration of neurotransmitter precursors on cognition. Upon administration, precursors are assumed to be converted into their end-products, thus increasing neurotransmitter levels and consequently, influencing cognitive function. For example, tyrosine and tryptophan are two amino-acid precursors of monoamine neurotransmitters that have been demonstrated to provide neuromodulatory effects. Tyrosine is a precursor of dopamine and norepinephrine, and its administration has been shown to modulate, amongst others, working memory^1, 2^ and Stroop performance^3^ (for a review, see ref. 4). Tryptophan is a precursor of serotonin (5-HT) and its administration has been shown to affect social behaviour and mood (for reviews, see refs 5–7), as well as improve or impair cognitive function depending on the individual’s mood and stress level due to mild sedation^5^. While neuromodulatory effects of tyrosine and tryptophan have attracted substantial research attention, the amino-acid precursor glutamine (Gln) has not been well researched as a potential neuromodulator of cognitive function despite being the precursor of glutamate (Glu) and γ-aminobutyric acid (GABA)^8^, which are the main excitatory and inhibitory neurotransmitters, respectively, within the brain^9^. After absorption into the circulatory system, Gln is able to pass through the blood-brain barrier^10^ upon which it then increases Glu and GABA levels in the brain^11^. Glu and GABA play critical roles in shaping cortical excitability and synaptic plasticity^12–17^. Because of their effects on cortical excitability, levels of Glu and GABA are implicated in, amongst others, response selection and inhibition^18–20^, impulsivity^21^, and error detection and response conflict monitoring^22^. Notably, cortical excitation facilitates the adjustment of synaptic strength via NMDA-receptor-driven long-term potentiation (LTP)^23^, thereby implicating Glu and GABA in learning as well. Given that Gln administration, via central changes in Glu and GABA levels, has the potential to alter cortical excitability and thus response selection and inhibition behaviour, it seems appropriate to consider the effects of Gln administration on sequence learning. Sequenced actions heavily rely on response selection processes^24^ and the acquisition of sequence patterns is associated with increases in cortical excitability^25^. Because sequenced actions are fundamental to most everyday tasks in humans^26^, it is important to investigate the potential neuromodulatory effects provided by Gln administration.

### Glutamate and GABA

As the primary excitatory and inhibitory neurotransmitters in the brain, higher levels of Glu and GABA respectively increase and decrease cortical excitability. It is hypothesized^18–20^ that increased cortical inhibition due to high GABA levels can sharpen task-relevant representations in the cortex and inhibit competing responses, thereby facilitating response selection and inhibition processes. It could then be argued that increased cortical excitation due to high Glu levels might have the opposite effect by facilitating activation of competing responses, thus increasing the time necessary to resolve response selection processes and impairing accuracy of these processes^20^. This model of the roles of Glu and GABA in response selection is supported by studies that directly assessed brain neurotransmitter levels using magnetic resonance spectroscopy (MRS), albeit with a particular focus on GABA. For example, individual differences in regionally-specific GABA concentration have been shown to predict motor decision speed in a saccade distractor task^27^, with higher levels predicting faster response initiation to a target in the face of distractors. Conversely, higher striatal GABA concentration has been associated with overall faster responses^28^ and higher accuracy^29^ in the Simon task. Furthermore, higher GABA concentration, in particular in airplane pilot trainees, has been associated with a more serial as opposed to parallel action cascading strategy, which has been argued to indicate more efficient action control^30^. Lastly, one study using MRS to assess the balance between Glu and GABA, rather than their individual levels, indicated a higher Glu-to-GABA ratio is associated with increased selection costs and slower reaction times in language production tasks^20^. In sum, there is converging support for the idea that increased GABA facilitates response selection via reduced cortical excitability, whereas increased Glu impairs response selection via heightened cortical excitability.

With respect to learning, studies have indirectly examined GABA and Glu by assessing the behavioural effects of a history of concussive injuries, which is thought to lead to accumulation of brain GABA and consequently stronger intracortical inhibition. This has important implications for learning, as excitation of the cortex facilitates LTP-driven learning via activation of NMDA receptors^23^. Consistent with reduced LTP due to higher GABA levels, previously-concussed athletes demonstrated reduced synaptic plasticity and less implicit motor sequence learning in a serial reaction time (SRT) task when compared to unconcussed teammates^31^. A follow-up study demonstrated that older concussed athletes had greater age-related decreases of Glu and that Glu concentration was positively related to motor sequence learning in the SRT task^32^. These findings are consistent with the important role of the primary motor cortex (M1) in sequence learning^33^ and the fact that M1 is sensitive to Glu and GABA^34–36^. In sum, they suggest sequence learning would benefit from increased excitation and suffer from increased inhibition of the cortex.

### The present study

Despite the critical roles of Glu and GABA in response selection and cortical excitability, we are not aware of any studies with healthy adults that have focused on the effects of Gln administration on these processes. Because of this lack of previous studies and the fact that Gln is the precursor to both Glu and GABA, which are hypothesized to have opposite effects on response selection and learning, it is difficult to establish *a priori* the direction in which Gln administration modulates performance. This is further compounded by the aforementioned finding that not just individual Glu and GABA levels but also the relative balance between the two determines performance^20^ and it remains unclear in favour of which neurotransmitter Gln would modulate this balance, if at all. Nevertheless, the direction of our results can tentatively suggest how the Glu-to-GABA ratio is modulated. As indicated by the previously discussed findings, increased GABA facilitates response selection but impairs motor sequence learning. Thus, improved response selection and/or impaired learning performance following Gln administration could be indicative of an increase in GABA level (compare^31^). The opposite results, i.e. decrements in response selection and/or enhanced learning performance, would then be consistent with an increased Glu level (compare^32^).

To investigate the effect of low dose Gln (2.0 g) on sequence learning we utilized the SRT task^37–39^, performance on which has been related to Glu and GABA levels (e.g. refs 31, 32). It represents a simple 4-choice reaction time task and thus involves response selection, inhibition and error detection processes that may be sensitive to a Gln-induced manipulation of Glu and/or GABA levels. The response sequence can be varied randomly, in which case participants can rely solely on the stimulus for selecting the appropriate response and have a 33% chance of guessing the correct response, given that there are no immediate response repetitions. As such, random blocks are particularly stimulus-oriented. However, the task also includes blocks with an embedded, second-order conditional (SOC) sequence that allows (unconscious) anticipation of the correct response and potentially induces a shift from stimulus-based to plan-based control^40^. The implicit learning of the SOC sequence is typically reflected in a gradual decrease in response latency, and modulation of this decline in response latency would indicate a potential influence of Gln on motor sequence learning. Contrasting results from stimulus-oriented, random blocks with those from SOC blocks can shed light on a possible differential effect of Gln on stimulus-based versus plan-based action control.

## Results

To assess the effect of Gln on processes associated with response selection and inhibition, one group of participants was administered 2.0 g Gln (*N* = 48) and another group received a neutral placebo (*N* = 43). Groups were then compared on response error percentage (REP) and mean reaction time (MRT) in the SRT task.

### Response error percentage

**Figure 1:**
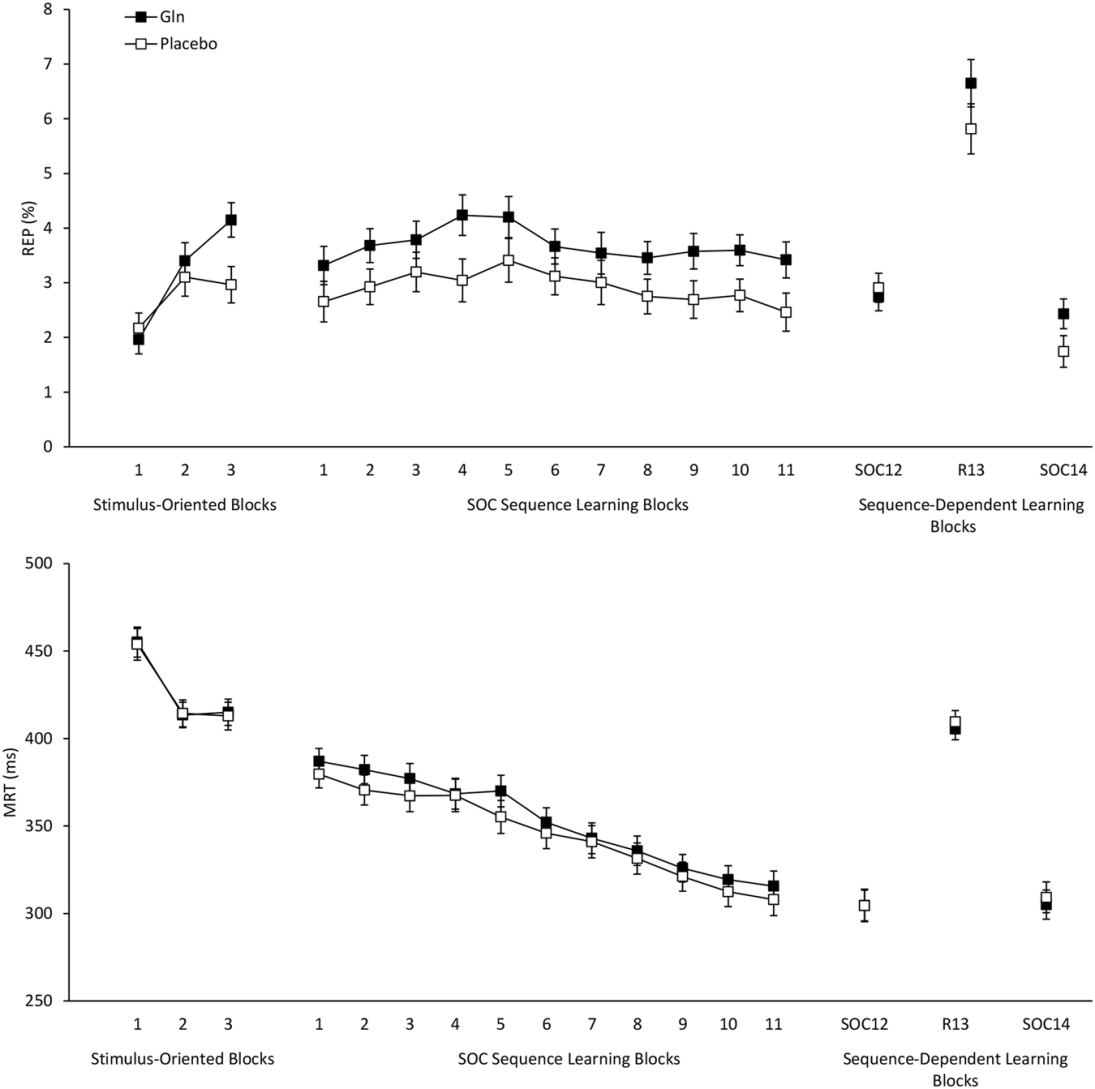
Mean REP (top panel) and MRT (bottom panel) as a function of block and group (Gln vs. placebo). ‘SOC’ refers to an SOC sequence block and ‘R’ refers to a random sequence block. Bars represent standard error of the means.

REP results are illustrated in Fig. 1 (top panel). In the first three stimulus-oriented blocks, repeated measures ANOVA indicated a non-significant effect of Group, *(p* = 0.21), but a significant effect of Block, *F*(2, 178) = 20.6, *p* < 0.001, partial *η* ^*2*^ = 0.19 and a significant Group × Block interaction, *F*(2, 178) = 4.1, *p* = 0.02, partial *η* ^*2*^ = 0.04. REP for the Gln group in stimulus-oriented block 1 (*M* = 1.96, *SD* = 1.53) and block 2 (*M* = 3.40, *SD* = 2.50) was not significantly different from REP for the placebo group in block 1 (*M* = 2.17, *SD* = 2.06) and block 2 (*M* = 3.10, *SD* = 2.02) (*p* = 0.58, 0.53). However, in block 3 REP was significantly higher (*p* = 0.012) in the Gln group (*M* = 4.15, *SD* = 2.37) than the placebo group (*M* = 2.97, *SD* = 1.97). To further explore the effect of Gln on REP we divided error trials in the first three stimulus-oriented blocks according to whether current error trial and the preceding trial required the same or a switched hand to make the response. REP was then submitted to repeated measures ANOVA with the factors Group and Hand Switch (same vs. switched hand). This revealed no significant interaction between Group and Hand Switch nor a three-way interaction involving Block, both *p* > 0.490. An additional analysis with the factors Group and Switch Direction (left-to-right vs. right-to-left) revealed no significant interaction between Group and Switch Direction nor a three-way interaction involving Block, both *p* > 0.731.

For SOC sequence blocks 1–11, repeated measures ANOVA indicated a significant effect of Group, *F*(1, 89) = 4.7, *p* = 0.03, partial *η* ^*2*^ = 0.05, and Block, *F*(10, 890) = 2.3, *p* = 0.01, partial *η* ^*2*^ = 0.03, while there was no significant Group × Block interaction, *(p* = 0.97). Across these blocks, the Gln group had significantly higher REP (*M* = 3.67, *SD* = 2.55) than the placebo group (*M* = 2.91, *SD* = 2.04). REP in blocks 4 and 5 was significantly higher than in block 1 while REP in block 11 was significantly lower than block 3–5 (all p < 0.05).

To further explore the effect of Gln on REP we divided error trials in SOC blocks 1–11 again according to whether the current error trial and the preceding trial required the same or a switched hand to make the response. The total number of errors per SOC block was then submitted to repeated measures ANOVA with the factors Group and Hand Switch. This revealed a significant effect of Group *F*(1, 89) = 4.34, *p* = 0.04, *η* ^*2*^ = 0.046 and Hand Switch, *F*(1, 89) = 123.1, *p* < 0.001, *η* ^*2*^ = 0.58, as well as a significant Group × Hand Switch interaction, *F*(1, 89) = 4.5, *p* = 0.037, *η* ^*2*^ = 0.048. Post-hoc comparisons indicated a significantly higher total amount of errors on trials requiring a switch of hands in both the Gln (*M* = 43.9, *SD* = 22.5 vs. *M* = 18.9, *SD* = 10.0) and placebo (*M* = 34.0, *SD* = 17.4 vs. *M* = 17.0, *SD *= 11.2) groups, both *p*s < 0.001. Importantly, the Gln group demonstrated significantly more switch hand errors (*p* = 0.023) than the placebo group whereas the groups did not differ on same hand errors (*p* = 0.406). Exploring these results yet further by dividing switch trials in those requiring a left-to-right or right-to-left switch revealed significant effects of Group, *F*(1, 89) = 5.4, *p* = 0.023, *η* ^*2*^ = 0.057 and Switch Direction, *F*(1, 89) = 55.6, *p* < 0.001, *η* ^*2*^ = 0.384, indicating significantly more errors on a left-to-right (*M* = 23.1, *SD* = 12.7) than right-to-left switch (*M* = 16.3, *SD* = 9.7), but this effect was not modulated by Gln as revealed by a nonsignificant Group × Switch Direction interaction, *p* = 0.658. In sum, Gln increased response errors when the hand required to carry out the target response on the current trial was not the hand used on the preceding trial and this effect was independent of the direction of the switch. To exclude the possibility this effect is driven by a group difference in amount of left-handed participants (see Table 1), we again analysed the effects of Group and Hand Switch on REP after excluding all left-handed participants. The Group × Hand Switch interaction remained significant and in the same direction, *F*(1, 80) = 4.4, *p* = 0.039, *η* ^*2*^ = 0.052, thereby excluding a potential confounding of the effect by group differences in handedness.

**Table 1:** Group characteristics.

	Gln	Placebo	*p*
N, Total	48	43	
N, Male:Female	14:34	13:30	0.912
N, Right:Lefthanded	40:8	42:1	0.022
Age, years *M (SD)*	20.5 (2.5)	20.6 (2.5)	0.904
Weight, kg *M (SD)*	66.1 (7.7)	65.1 (7.6)	0.531
BMI, kg/m^2^ *M (SD)*	21.6 (2.5)	21.9 (2.2)	0.632
Sleep, hours *M (SD)*	7.3 (1.3)	6.8 (1.2)	0.086

To assess sequence dependent learning we compared REP in the 12^th^ SOC block to the 13^th^ random block and the 14^th^ SOC block. Repeated measures ANOVA indicated a significant effect of Block, *F*(2, 178) = 128.1, *p* < 0.001, partial *η* ^*2*^ = 0.59, while the Group main effect (*p* = 0.21) and the Group × Block interaction, *(p* = 0.16) were not significant. REP in block 13 (*M* = 6.25, *SD* = 3.02) was significantly higher than block 12 (*M* = 2.82, *SD* = 1.74, *p* < 0.0001) and block 14 (*M* = 2.11, *SD* = 1.90, *p* < 0.0001). REP in block 12 was also significantly higher (*p* < 0.01) than in block 14. Additional analyses revealed no significant interaction between Group and Hand Switch, *F*(1, 89) = 0.22, *p* = 0.642, or between Group and Switch Direction (left-to-right vs. right-to-left), *F*(1, 89) = 0.13, *p* = 0.722.

### Mean reaction time

MRT results are presented in Fig. 1 (bottom panel). In each of three analyses, there was only a significant effect of Block in the three stimulus-oriented blocks, *F*(2, 178) = 89.2, *p* < 0.001, partial *η* ^*2*^ = 0.50, in SOC blocks 1–11, *F*(10, 890) = 104.4, *p* < 0.001, partial *η* ^*2*^ = 0.54, and when comparing SOC block 12 to random block 13 and SOC block 14, *F*(2, 178) = 321.4, *p* < 0.001, partial *η* ^*2*^ = 0.78. In contrast, no significant main effects of Group or Group × Block interactions were obtained (all *p* > 0.53). In the stimulus-oriented blocks, MRT was significantly higher in block 1 (*M* = 454.51, *SD* = 59.02) than block 2 (*M* = 413.86, *SD* = 50.19, *p* < 0.0001) and block 3 (*M* = 413.99, *SD* = 51.82, *p* < 0.0001) while MRT did not significantly differ between blocks 2 and 3 (*p* = 0.99). For SOC sequence learning blocks, MRT was significantly lower as learning progressed across blocks 1 to 11. For sequence-dependent learning assessment blocks, MRT in block 13 (M = 396.18, SD = 34.41) was significantly higher than block 12 (M = 299.69, SD = 56.10, p < 0.0001) and block 14 (M = 302.16, SD = 49.80, p < 0.0001). MRT in block 12 was not significantly different (p = 0.37) than MRT in block 14. None of the stimulus-oriented blocks, SOC sequence learning blocks or sequence-dependent learning blocks involved a significant Group × Hand Switch interaction, all *p* > 0.31, or a significant Group × Switch Direction interaction, all *p* > 0.63. In sum, Gln did not affect MRT in any of the SRT blocks.

### Sequence recall

The frequency of participants reporting explicit awareness of a sequence pattern was not significantly different between Gln (“Yes” = 36, “No” = 12) and placebo (“Yes” = 34, “No” = 9) groups *X* ^*2*^(91) = 0.21, *p* = 0.65. The number of sequence chunks recalled did not significantly differ between Gln (M = 1.04, SD = 0.54) and placebo groups (*M* = 1.00, *SD* = 0.56, *p* = 0.72). Similarly, the average length of sequence chunks recalled did not significantly differ between Gln (M = 4.65, SD = 2.70) and placebo groups (*M* = 4.91, *SD* = 2.87, *p* = 0.66). In sum, Gln did not seem to affect measures of sequence recall.

## Discussion

The present paper is one of the first to report proof-of-principle that the amino-acid Gln, the precursor of the brain’s main excitatory neurotransmitter Glu and inhibitory neurotransmitter GABA^8, 9^, modulates cognitive function related to response selection but not sequence learning. Specifically, Gln administration led to an overall increase in response selection errors in both stimulus-oriented and SOC sequence-learning blocks of an SRT task without affecting sequence-dependent learning or sequence recall, suggesting Gln affected primarily stimulus-based rather than plan-based control^40^. More specifically, Gln impaired performance when the hand required to carry out the target response differed on the current and preceding trial, indicating Gln primarily affected the laterality of response selection processes. This raises the possibility that Gln, via a presumed increase in Glu, enhanced the lateral motor activation associated with the most recent target response, which presented conflict when the next trial required the other hand to press the correct key. This notion is indirectly supported by findings on GABA and the Simon task^28, 29^ that suggest increased cortical inhibition benefits processing of laterality of responses. The opposite, that is an impairment in processing the laterality of responses, might then be expected when cortical inhibition is reduced by Glu. Consistent with this idea, heightened cortical excitability has been hypothesized to account for impaired response selection by facilitating activation of competing responses^20^. Furthermore, it is interesting to note the increase in response selection errors emerged only after the first two (stimulus-oriented) blocks, indicating Gln might have affected performance only after initial task familiarization when participants might start responding with less deliberation and instead perform more automatically. On the other hand, the Gln effect seems to have worn off near the end of the task, suggesting that at this point Gln may have been metabolized down to levels that no longer influenced behaviour or participants in the Gln condition could overcome the effect on response selection with sufficient training. It is also important to note the Gln-induced increase in response errors was not due to a speed-accuracy trade-off, as response latencies were unaffected. In sum, the present study provides first evidence indicating Gln administration can modulate cognitive-behavioural performance by enhancing cortical excitability.

Whereas Gln seems to have affected response selection processes in both stimulus-oriented (random) and SOC blocks, its effects did not seem to extend to sequence-dependent learning. This might tentatively indicate that LTP-driven plasticity was not altered despite the presumed increase in cortical excitation. For now we can only speculate on the reason for this selectivity. First, it might be our Gln dose of 2.0 g was too low to induce changes in synaptic plasticity. Previous studies that mainly focused on Gln’s positive effects on gastrointestinal function administered daily doses of 10 g or more^41–43^, which exceeds the average daily intake of 3–6 g Gln^44^ and is 5 or more times our dose of 2.0 g. It remains unclear whether a higher dose could lead to a more pronounced and longer-lasting cognitive response, hence future studies might systematically vary Gln dose to clarify this issue. Second, Gln might have affected response selection but not sequence-dependent learning because of regional specificity, in line with reports of regionally-specific effects of GABA^12, 21, 27^. Although this remains speculative, perhaps Gln at a dose of 2.0 g primarily affected response conflict and impulsivity in the prefrontal cortex without affecting sequence-dependent learning mediated by motor area M1^33^. Future studies could employ MRS to assess whether Gln has dissociable effects on Glu and GABA levels in different regions. Third, in the present study three random, stimulus-oriented blocks were always presented before twelve SOC blocks. This order of presentation might have predisposed participants to stimulus-based rather than plan-based control and discouraged sequence learning in SOC blocks, limiting the possibility of finding an effect of Gln on plan-based control. Hence, whereas this study presented the random blocks *after* Gln administration and immediately before starting the SOC blocks, future studies may wish to present the first random blocks *before* Gln administration to render them more as task familiarization, or systematically vary the order in which the blocks are presented.

The lack of previous studies with Gln and cognitive-behavioural performance made it difficult to predict *a priori* the direction in which Gln would enhance performance. However, the present results may form a basis for novel hypotheses that can be tested in the future and thereby provide converging evidence that Gln modulates performance via increased cortical excitability. For example response inhibition, i.e. the ability to withhold prepotent responses, seems to benefit from increased cortical inhibition due to increased GABA levels^45, 46^. Similarly, higher GABA levels have been associated with less impulsivity^21^. This suggests the opposite, that is an impairment in response inhibition and increase in impulsivity, might occur when cortical excitability is enhanced due to Gln-induced increases in Glu levels. Hence a study showing Gln reduces response inhibition efficiency and/or increases impulsivity would converge on the idea Gln enhances cortical excitability.

The present study employed a between-subjects design because asking participants whether they noticed a response sequence in the SRT task is likely to affect subsequent task performance. Because of the between-subjects design, one might argue our results are simply due to baseline group differences in response selection efficiency rather than due to the Gln administration. Although we argue this is unlikely with our sample size that is larger than in most amino-acid precursor studies, this is not to say such alternative explanations of our data are impossible. Therefore, future studies may wish to exclude the possibility of pre-existing group differences by i) using tasks that allow for a within-subjects design, ii) assessing baseline response selection performance and iii) taking pre and post-administration measurements of cortical excitability, for example by using motor-evoked potentials (MEP) to assess both pre-existing differences and Gln-induced changes in cortical excitability. If MEP measurements confirm enhanced excitability of the cortex after Gln administration and if this change would correlate with the individual frequency of response selection errors, that would provide strong support for the mechanism of action hypothesized in the present study.

It may also be interesting for future studies to consider the role of individual differences in the balance between cortical Glu and GABA levels, as variability in this balance rather than the individual neurotransmitter levels has previously been shown to predict individual differences in response selection efficiency^20^. Although the present study suggests Gln administration at a group level enhanced this balance in favour of Glu, it seems plausible individual response to Gln might be predicted by the pre-existing Glu/GABA balance, with Gln perhaps having more pronounced or even opposite effects in individuals with a balance highly in favour of Glu or GABA.

Lastly, it is important to consider that the effect of Gln reported here seems to apply to sequential motor control but not sequence learning, as Gln affected performance similarly in the stimulus-oriented and SOC blocks of the SRT task. However, the present version of the SRT task includes relatively few stimulus-oriented blocks, which limits the assessment of sequential motor control separately from sequence learning. To disentangle and separately investigate these processes, future research should aim to balance the amount of stimulus-oriented and SOC blocks^47^.

To conclude, the present study is the first to investigate Gln administration in healthy adults in relation to response selection and sequence learning performance. Results show Gln impairs response selection but does not alter sequence learning, suggesting an increase in cortical excitability without affecting synaptic plasticity. As such, despite the critical roles of Glu and GABA in motor learning, this study finds no evidence for an effect of their precursor on sequence learning performance, but does present first evidence that Gln modulates sequential motor control processes.

## Methods

### Participants

A total of 91 students from Leiden University were recruited to participate for money or course credit. Using a double blind, placebo-controlled design, participants were randomly assigned to receive either Gln (*N* = 48) or a neutral placebo (*N* = 43). See Table 1 for group characteristics. The groups did not differ in terms of gender distribution, age, weight, BMI, or hours of sleep the night before the study, but the Gln group did contain significantly more left-handed individuals.

Study participation eligibility criteria were based on previous studies on neuromodulation from our lab^1, 48–50^. Specifically, interested individuals were screened for cardiac, hepatic, renal, neurologic or psychiatric disorders, and medication (except oral or implanted contraceptives) or recreational drug use, and those who reported any of these conditions were not eligible to participate. Females were only eligible to participate if they used either oral or implanted hormonal contraception, to reduce the impact of fluctuating estrogen-dopamine interactions on our results^51–53^.

Prior to participation informed consent was obtained from all participants. The study conformed to the ethical standards of the Declaration of Helsinki and the protocol was approved by the local ethical committee (Leiden University, Institute for Psychological Research).

### Glutamine administration

All doses were prepared and coded by a researcher not involved in running the study, to blind the experiment leader to the administered dose. Participants received either 2.0 g Gln or 2.0 g microcrystalline cellulose, a neutral placebo, dissolved in 400 mL of orange juice. Given the lack of prior studies on Gln administration and cognition, this dose was based on previous studies with tyrosine in our lab that showed reliable effects^1, 54, 55^. The dose of 2.0 g is safe and less than the normal daily intake of approximately 3–6 g Gln from protein^44^ and far less than in studies on Gln and gastrointestinal function that administered 10 g or more daily^41–43^.

### Serial reaction time task

To assess response selection and sequence learning participants performed a standard SRT task^37–39^ presented using E-Prime 2.0 software (Psychology Software Tools, Inc., Pittsburgh, PA, USA). In this task four horizontally-aligned empty squares were presented in the centre of the screen. On each trial one of the squares turns red and the participant must press a corresponding button on the QWERTY keyboard (from left to right: V, B, N, M) using the index and middle fingers of the left (V, B) and right (N, M) hand. An error sound is presented if the wrong button is pressed, along with the Dutch words “Verkeerde toets!” (“Wrong button!”). Reaction time (RT) is measured in milliseconds as the latency in the key press to the stimulus and if RT exceeds 3,000 ms, the Dutch words “Te langzaam!” (“Too slow!”) are presented. Following the response, the four empty squares appear for a 50 ms response-stimulus interval before the next stimulus is presented. Participants were instructed that accuracy and response speed were equally important in the task. Participants first completed three random sequence blocks, then twelve SOC sequence learning blocks in which responses followed a 12-item SOC sequence (VBVNMBNVMNBM^56^), which was cycled through ten times in each block. To determine sequence dependence or the serial effect as opposed to general practice effects, a random-ordered block was inserted followed by a final block that re-introduced the SOC sequence. The sudden introduction of a random response sequence likely interferes with the anticipation of responses in a plan-based action control style, requiring an abrupt shift to stimulus-oriented control. Hence RT and response errors are expected to sharply increase in the random block^57^ but performance is expected to recover when the sequence is reintroduced in the final SOC block. All blocks contained 120 trials and after completion of each block performance feedback indicating number of errors and mean RT was presented followed by a 30 s rest interval. Following completion of the final block, participants were asked to respond “Yes” or “No” to a question that asked if they noticed a pattern in the responses at any point of the task to determine explicit awareness of the serial sequence. When answering “Yes”, participants were then asked to use the response keys to produce one cycle of the 12-item sequence as a recall test.

### Procedure

Participants entered the lab between 9:00 and 10:00 to be tested individually, having fasted overnight (compare^1, 48^). Informed consent was obtained, after which they consumed 2.0 g of either Gln or placebo dissolved in 400 mL orange juice. Afterwards, apples and oranges were offered to prevent strong hunger. Although Gln has been shown to increase *in vitro* rat brain Glu and GABA levels as soon as 15 minutes^11^, in line with previous studies on precursors of neurotransmitters, testing commenced precisely one hour after Gln or placebo administration to allow time for plasma and brain Gln levels to increase (compare^1, 48, 55^). Participants then performed the SRT task, which took approximately 30 minutes. Lastly, participants were debriefed on the nature and hypotheses of the study and were compensated and thanked for their participation.

### Analysis

For each participant, REP was determined based on the number of error trials (incorrect key press) as a percentage of the total number of trials in each block. MRT was then calculated for each participant and each block, after removing error trials as well as trials with outlier RT (1.2%) based on RT that was more than 3 standard deviations above the individual’s overall mean for correct trials. Participant REP and MRT were then submitted to separate repeated measures ANOVA that i) compared performance between Gln and placebo Groups in the first three stimulus-oriented Blocks, ii) compared sequence learning between Gln and placebo Groups in SOC Blocks 1–11, and iii) compared sequence-dependent learning in Gln and placebo Groups across Blocks 12 (SOC), 13 (random) and 14 (SOC). Although others^39, 57^ proposed averaging performance over SOC Blocks 12 and 14 before comparison with random Block 13, performance in Block 14 might still suffer from interference by the previous random block. As such, we argue that comparing all three blocks can provide a clearer picture of how performance is affected by the introduction of the random block. Explicit sequence awareness frequency was compared between Gln and placebo groups using Χ^2^ analysis. Sequence recall was analysed based on a response chunking approach^58–60^. The presence of chunks of the training SOC sequence was determined for each participant with chunk lengths ranging between 4 to 12 items. The probability of entering the smallest chunk length, a 4-item chunk, by chance was calculated as 11% (0.33 × 0.33) given that there are no consecutive repetitions in the SOC sequence structure^61^. To identify any matches between the participant’s recalled sequence and the target sequence, the participant’s sequence was divided into chunks made up of between 4 and 12 items. These chunks could commence with any sequence item, with the condition that the end of the sequence could not be extended to the initial sequence items since the participant’s chunk needed to be contiguous. The target sequence was also divided into chunk lengths of between 4 and 12 items, however, here these chunks could start with any item in the sequence and continue on to include items at the beginning of the sequence. Continuing the chunks past the end of the SOC sequence reflects the repeating nature of the sequence, meaning the participant could have treated the commencement of a chunk at any point of the repeated sequence. Performance on chunk recall of the sequence was based on the number of matched chunks and mean length of the matched chunks for each participant. Only the longest chunk was recorded as a match and matched chunks were only recorded once in the event the participant repeated the same chunk. As the 12-item sequence recall allows for 9 possible 4-item chunks, a participant would be expected to recall approximately 1 valid 4-item chunk by chance (9 × 0.11). Participant’s recalled chunk count and mean chunk length were separately submitted to ANOVA for Group comparisons. All repeated measures analyses use Greenhouse-Geisser correction when the sphericity assumption was violated and all post-hoc comparisons use Fisher’s LSD adjustment. For all tests a significance threshold of 0.05 was adopted.

Random ordering of responses in the first three stimulus-oriented blocks and random Block 13 could have introduced group differences in the number of reversal trials resulting in MRT performance artefacts^47, 56^. A reversal trial occurs when the third trial of any three consecutive trails involves the same target response as the first trial (e.g., VBV^47^). With respect to the number of reversal trials in the first three stimulus-oriented blocks, there was a non-significant effect of Group (*p* = 0.21) and a non-significant Group × Block interaction (*p* = 0.13). In addition, the number of reversal trials in Block 13 did not significantly differ between Groups (*p* = 0.12). In stimulus-oriented blocks, MRT was significantly longer in reversal trials than in non-reversal trials, *F*(1, 89) = 145.8, *p* < 0.0001, partial *η* ^*2*^ = 0.62, however, there was a non-significant Group × Reversal Trial interaction (*p* = 0.68) and a non-significant Group × Reversal Trial × Block interaction (*p* = 0.61). In SOC blocks, MRT was significantly longer in reversal trials than in non-reversal trials, *F*(1, 89) = 24.5, *p* < 0.0001, partial *η* ^*2*^ = 0.22, however, there was again a non-significant Group × Reversal Trial interaction (*p* = 0.56) and a non-significant Group × Reversal Trial × Block interaction (*p* = 0.89). This indicates any group differences in these blocks are not confounded by differences in the number of reversal trials.
